# La maladie de Lobstein dans sa forme historique

**DOI:** 10.11604/pamj.2014.19.293.5444

**Published:** 2014-11-17

**Authors:** Karima Atarraf, Moulay Abderrahmane Afifi

**Affiliations:** 1 Service d'Orthopédie Pédiatrique, CHU Hassan II, Faculté de Médecine et de Pharmacie, Université Sidi Mohammed Ben Abdullah, Fès, Maroc

**Keywords:** Maladie de Lobstein, déformation, épaississement des corticales, Lobstein disease, deformation, thickening of cortical

## Image en medicine

Jeune fille âgée de 14 ans, présentant une déformation de la cuisse droite évoluant depuis l’âge de 2 ans et d'aggravation progressive. L'examen clinique a trouvé des sclérotiques bleus et une déformation de la cuisse droite avec une inégalité de longueur de 5 cm. La radiographie de la cuisse a objectivé une déformation historique du fémur, avec épaississement des corticales et comblement du canal médullaire avec une coxa vara (A). Le diagnostic d'ostéogenèse imparfaite a été retenu et la patiente a bénéficié d'un enclouage télescopique après deux ostéotomiesn (mini abord) (B). L'ostéogenèse imparfaite ou la maladie des os verres ou encore maladie de lobstein est une maladie génétique rare caractérisée par une importante variabilité de l'expression du génotype lié à la grande variabilité des mutations des gènes responsables de la synthèse des deux chaines de collagène type I; il en résulte une fragilité osseuse et une anomalie de la minéralisation de la matrice de l'os. Si le traitement médical basé sur les biphosphonates représente une approche thérapeutique innovante; la prise en charge orthopédique et physiothérapique; dont la fiabilité et l'efficacité ont été établi pour le fémur; reste la base du traitement, car il permet une protection effective et prolongée. Reste à savoir que l’âge est un élément épidémiologique et pronostique important dans la prise en charge, ceci pour éviter l'aggravation des déformations qui va retentir sur la mobilité et la croissance en longueur du membre étant le cas de notre patiente.

**Figure 1:**
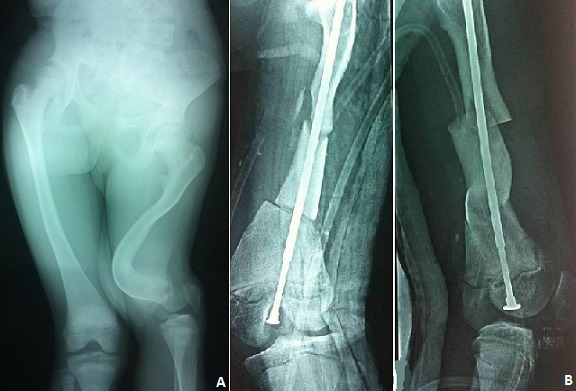
A) radiographie des deux cuisses objectivant une déformation historique du fémur, avec une angulation de 90°, épaississement des corticales et comblement du canal médullaire avec une coxa vara; B) résultat final après double ostéotomie de réaxation et enclouage centro-medullaire

